# The Value of Microbes in Cancer Neoantigen Immunotherapy

**DOI:** 10.3390/pharmaceutics15082138

**Published:** 2023-08-14

**Authors:** Junrui Tian, Jian Ma

**Affiliations:** 1 NHC Key Laboratory of Carcinogenesis and Hunan Key Laboratory of Cancer Metabolism, Hunan Cancer Hospital and the Affiliated Cancer Hospital of Xiangya School of Medicine, Central South University, Changsha 410013, China; 226511035@csu.edu.cn; 2 Cancer Research Institute and School of Basic Medical Science, Central South University, Changsha 410078, China; 3 Key Laboratory of Carcinogenesis and Cancer Invasion of the Chinese Ministry of Education, Hunan Key Laboratory of Nonresolving Inflammation and Cancer, Changsha 410078, China

**Keywords:** tumor neoantigen, cancer immunotherapy, microbes, cancer vaccines, immune checkpoint inhibitors

## Abstract

Tumor neoantigens are widely used in cancer immunotherapy, and a growing body of research suggests that microbes play an important role in these neoantigen-based immunotherapeutic processes. The human body and its surrounding environment are filled with a large number of microbes that are in long-term interaction with the organism. The microbiota can modulate our immune system, help activate neoantigen-reactive T cells, and play a great role in the process of targeting tumor neoantigens for therapy. Recent studies have revealed the interconnection between microbes and neoantigens, which can cross-react with each other through molecular mimicry, providing theoretical guidance for more relevant studies. The current applications of microbes in immunotherapy against tumor neoantigens are mainly focused on cancer vaccine development and immunotherapy with immune checkpoint inhibitors. This article summarizes the related fields and suggests the importance of microbes in immunotherapy against neoantigens.

## 1. Introduction

Tumor neoantigens can be summarized as substances with immunogenic properties that are specifically expressed by tumor cells. Due to their specific presence on tumor cells and the fact that the T-cells recognizing these antigens are not affected by central T-cell tolerance [1], neoantigens are considered ideal targets for cancer therapy [2,3]. The content about tumor neoantigens has been evolving, and with continuous research, many types of neoantigens have been identified. Tumor neoantigens can be divided into two categories: classical neoantigens and noncanonical neoepitopes [4]. Among these, classical neoantigens are derived from cancer-specific genetically hardwired alterations, including oncogenic missense mutations, frameshift mutations, splice sites, gene fusions, and long noncoding RNA-derived neoantigens. Noncanonical neoepitopes are not derived from genetic alterations, encompassing neoepitopes originating from alternative splicing, post-translational modifications, RNA editing, and aberrant mRNA translation [4,5]. Cancer immunotherapies developed against neoantigens have developed rapidly in recent decades [6]. The main therapeutic strategies are neoantigen vaccines [7,8], adoptive cell transfer (ACT) therapy [9,10], and immune checkpoint blockade therapy [11].

Microbes have been found to play an important role in cancer immunotherapies. Immunotherapy aims to trigger a specific anti-tumor response in cancer patients. The availability of specific neoepitopes in tumor cells, as well as the ability of these neoepitopes to effectively activate immune cells targeting such epitopes, are necessary for successful immunotherapy [2,12]. Numerous studies have shown that microbes play a regulatory role in the host immune system. They can modulate the host immune system by virtue of their immunogenic peptides or metabolites, activate the relevant immune cells, and enhance the effect of immunotherapy [13,14,15]. In addition, because microbes are small in size and simple in structure, researchers can achieve the modification of microbes more easily via genetic engineering and use them as tools in the development of neoantigen immunotherapy [16,17]. Using these modified microbes or their components, it is possible to design suitable delivery vehicles for neoantigen vaccines or drugs targeting neoantigens [18]. The judicious use of microbes has great potential in immunotherapy against tumor neoantigens.

Here, we reviewed the current research on tumor neoantigens and microbes and summarized the development of neoantigen identification. We discussed how microbes influence the mechanisms of neoantigen immunotherapy and hope to provide a better guideline for the application of microbes in neoantigen immunotherapy. We also summarized the importance and application results of microbes in cancer neoantigen vaccines and immune checkpoint blockade therapy in recent years. We hope this review can draw attention to the importance of microbes in neoantigen immunotherapy, advancing the development of therapeutic approaches.

## 2. The Development History of Neoantigen Identification

Since the discovery and isolation of the first tumor neoantigen, P91A, in a mouse tumor model by De Plaen’s team in 1988 [19], there has been continuous development regarding the identification of neoantigens. Due to technical limitations at the time, early neoantigen identification [20,21,22] was usually low-throughput and labor-intensive. For nearly twenty years, most neoantigens were identified by constructing cDNA libraries, overexpressing the selected neoantigen cDNAs and major histocompatibility complex (MHC) molecules in cell lines, co-culturing them with T cells, and finally determining the immunogenicity of the screened neoantigens by measuring the differentiation status of the T cells. At the same time, mass spectrometry is also being applied to the identification of neoantigens [23]. Human leukocyte antigen (HLA) molecules on the surface of tumor cells are first isolated, and then these peptides are analyzed by mass spectrometry to identify tumor neoantigens.

The period of rapid development of tumor neoantigen identification technology came with the birth of next-generation sequencing (NGS). In 2012, Hirokazu Matsushita et al. identified neoantigens in mouse sarcoma cells for the first time with the help of NGS approaches [24]. This technique identified mutant proteins expressed in patients’ tumor cells by whole exome sequencing analysis and then predicted candidate mutant T-cell epitopes [25]. Theoretically, by continuously optimizing the MHC prediction model, a large number of mutant neoantigens can be rapidly identified. On the basis of this theory, many prediction pipelines were subsequently developed. Examples include p-VAC-seq [26], which integrates tumor mutation and expression data and automates multiple antigen screening steps; PSSMHCpan [27], which can effectively predict the affinity of peptide binding to HLA class I alleles; DBTpred [28], which focuses on single amino acid residue mutations resulting in altered peptide-MHC binding affinity; and RBM-MHC [29], which improved predictions for rare alleles. In addition to these algorithms based on peptide affinity, there are also prediction methods that assess the prediction of immunogenicity based on the stability of the peptide-MHC complex [30,31].

Most of these algorithms predict neoantigens arising from genetic mutations while ignoring neoantigens produced by other possibilities. Recently, identification methods for neoantigens arising from these non-mutational alterations have been gradually developed. For instance, identification of neoantigens arising from alterations in extracellular regions of membrane proteins [32], gene fusions [33], single nucleotide variations (SNVs), Indels, and gene fusions by analysis of original sequencing data [34,35]. The computer prediction of MHC class-II binding epitopes is more complex compared to the prediction of MHC class-I binding epitopes because the binding properties of the peptide-binding groove of these molecules are less stringent [36]. In recent years, a series of prediction pipelines for predicting MHC-II binding epitopes have been developed—for example, MAPTAC [37] and FIONA [38]. With the continuous maturation of tumor neoantigen identification technology, a large number of neoantigens have been identified. Researchers have also built more complete neoantigen databases on this basis [39,40,41], and these abundant neoantigen resources have, in turn, contributed to the accuracy of neoantigen prediction [42].

In addition to computerized prediction models, mass spectrometry (MS) to identify neoantigens has been widely used with the advent of NGS technology [43]. With high mass resolving power, MS can identify typical and non-typical antigens from the MHC-binding peptides, reducing the false–positive rate [44]. For example, with the help of gene sequencing and MS analysis, researchers identified new epitopes of tumor antigens in the mouse tumor model [45,46]. Specific neoantigens have also been identified in human tumor tissue samples by MS technology [47]. The coupling of liquid chromatography (LC) with MS, such as LC–tandem MS (LC–MS/MS) [48] and nano-ultra-performance LC coupled to high-resolution MS (nUPLC–MS/MS) [49], has expanded the coverage of the MHC peptidome and improved the sensitivity of MS, which has more advantages in neoantigen identification. In recent years, there has been continuous research combining mass spectrometry techniques with computerized prediction models to develop efficient and accurate prediction channels. For example, NetMHCpan-4.0 [50], which is commonly used in neoantigen identification, can integrate the MHC-peptide binding affinity (BA) datasets and MS-eluting ligand (EL) datasets into a single framework to train machine learning models and obtain superior prediction performance. Training prediction models using MS data greatly improves the specificity of HLA-peptide binding prediction algorithms [51,52].

Based on the evolving neoantigen prediction methods described above, many neoantigens have been identified. However, not all predicted neoantigens are immunogenic [53]. Only a small fraction of the mutated peptides identified by bioinformatics are immunogenic [54]. Some studies show that the quality rather than the quantity of neoantigens expressed by tumor cells could better predict clinical outcomes [55,56]. Neoantigens with homology to infectious disease-derived epitopes would be more immunogenic [55], which provides inspiration for subsequent identification of neoantigens.

**Figure 1:**
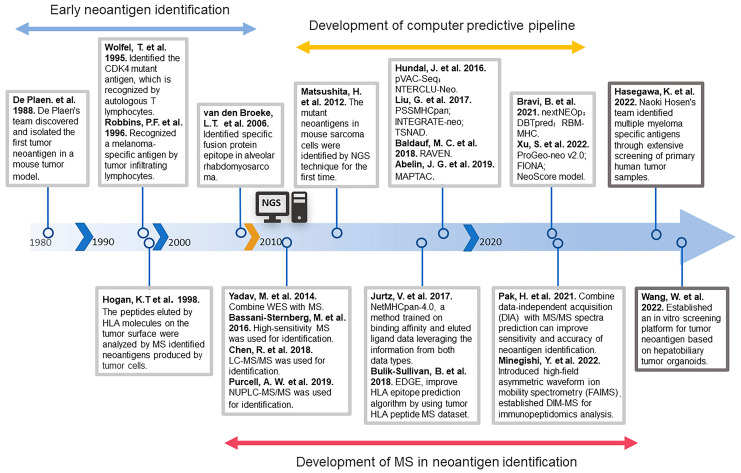
The history of the development of neoantigen identification [19,20,21,23,24,26,27,29,37,38,45,47,48,49,50,51,57,67,68,69,70,71].

With the in-depth study of neoantigens, many previously unnoticed neoantigens have been discovered [4], such as neoantigens arising from post-translational modifications and RNA editing. These neoantigens do not result from alterations at the gene level and cannot be identified by relying on sequencing technology or MS-based analysis. It is important to develop new identification methods with the help of new technologies. Recently, Naoki Hosen’s team identified a specific antigen for multiple myeloma through extensive screening of primary human tumor specimens and found that the specificity of this antigen is due to altered protein glycosylation [57]. This provides a new dimension for neoantigen identification. In recent years, with the maturation of glycoproteomics [58,59], some researchers have pointed out that glycoproteomics has great potential for future applications in the identification of cancer-specific antigenic epitopes formed by post-translational modifications of proteins [60,61,62]. Many studies have revealed that tumor cells exhibit unique glycoproteins on their surfaces [63,64]. Therefore, the integration of glycomics and glycoproteomics into neoantigen discovery platforms is of great significance for neoantigen identification [60]. To better identify immunogenic neoantigens, researchers have recently introduced tumor organoids into neoantigen identification. Organoids can better mimic the structure and function of in situ tumor cells in vitro [65,66]. Using this method, researchers characterized the HLA-class-I neoantigen landscape in hepatobiliary tumors, providing a practical strategy with a tumor organoid model for neoantigen peptide identification in personalized immunotherapy [67]. In conclusion, during the three decades of the development of tumor neoantigen identification, many prediction methods for neoantigens have been developed based on the continuous development of two major technologies—NGS and MS (Figure 1). Nowadays, the superficial neoantigen-based library has been basically tapped, so we need to use new means and methods to tap the potential neoantigen library.

## 3. Microbial and Tumor Neoantigens

### 3.1. Homology of Microbes and Tumor Neoantigens

Individuals are exposed to an environment full of various microbes, and tumors are not independent of the environment. Tumorigenesis is closely related to the contribution of pathogens in the environment—for instance, the common human papillomavirus (HPV) [72], the hepatitis B/C virus (HBV, HCV) [73], the Epstein–Barr virus (EBV) [74], and some cancer-inducing pathogenic bacteria such as *Helicobacter pylori* [75]. Meanwhile, some microbes have also been closely associated with cancer therapies [76]. In recent years, microbial relevance has been increasingly found in the study of tumor neoantigens [56,77,78,79]. Here, we describe the significance of microbes in tumor neoantigens. After nearly three decades of research, many neoantigens have been identified. However, not all of them are highly immunogenic. Searching for tumor neoantigens that are immunogenic and can activate T-cell responses is the key to targeted tumor therapy. Several reports have indicated that many neoantigens have been found to be homologous to microbial-derived peptides [56,77,78,80] (see Table 1). And such derived peptides usually have the same epitope core as the tumor neoantigen [81]. Furthermore, some studies have shown that neoantigens homologous to pathogenic antigens are more likely to be immunogenic than non-homologous neoantigens [82,83], which makes microbes very attractive in the field of tumor neoantigen research.

A major source of tumor neoantigens is the peptides encoded by viral genes [4]. These oncogenic viral-encoded molecules can be used to distinguish tumor cells from normal cells, exhibiting unique characteristics of tumor cells and being a major target for early neoantigen identification [84]. Viruses enter cells and use host cells to encode their own proteins. These peptides are presented on the surface of tumor cells and can be judged as non-self peptides by the host immune system, triggering an immune effect that specifically kills tumor cells. With the continuous enrichment of the Antigen Peptide Library, Ragone C. et al. recently screened all tumor-associated antigens (TAAs) in the literature and compared them with proteins from viral sequences in a homology search. They found 82 viral sequences homologous to TAAs, showing a high homology of sequence and structure between TAAs and viral sequences [85]. In some cases, this homology is striking, and this high-homology epitope does not resemble a random event. Researchers previously performed neoantigen prediction in patients with HCV-induced hepatocellular carcinoma (HCC) and found that mutated neoantigens showed >50% sequence similarity to pathogen-associated antigens (PaAs) [55]. Bioinformatics tools have also been developed to identify tumor peptides with high similarity to viral epitopes. This could help us better recognize tumor neoantigens that are homologous to pathogens [86].

In addition to the presence of neoantigen homologous sequences in pathogenic viruses, the bacterial community, especially the gut microbiota, is also considered a potential source for neoantigens. Most of the immune system’s exposure to the external environment occurs in the gastrointestinal tissue. The gut microbiome encodes more than 3 million genes in total, while the individual human genome has about 23,000 genes [87], so there is a high probability of homology between the two. The resident gut microbiota induces multiple reactions within the human body and is a great source of variant antigens [88]. A research team recently compared the homology of TAAs with the peptides from species of the *Firmicutes* and *Bacteroidetes phyla*, which together account for 90% of gut microbiota. They found a high degree of homology [89], which demonstrates the interactions between the microbiota colonizing the organism and tumor tissue. For instance, an epitope SVYRYYGL (SVY) was identified in the genome of the commensal bacterium *Bifidobacterium breve* (*B. breve*), which is homologous to the neoepitope antigen SIYRYYGL (SIY) expressed in B16 tumor models [77].

These studies suggest that homology between microbes and tumor neoantigens does not exist by chance (see Table 1). Additionally, the studies revealed that these highly similar sequences may harbor great potential for cancer therapy.

### 3.2. Immune Response Induced by Microbial Neoantigen Mimicry

**Figure 2:**
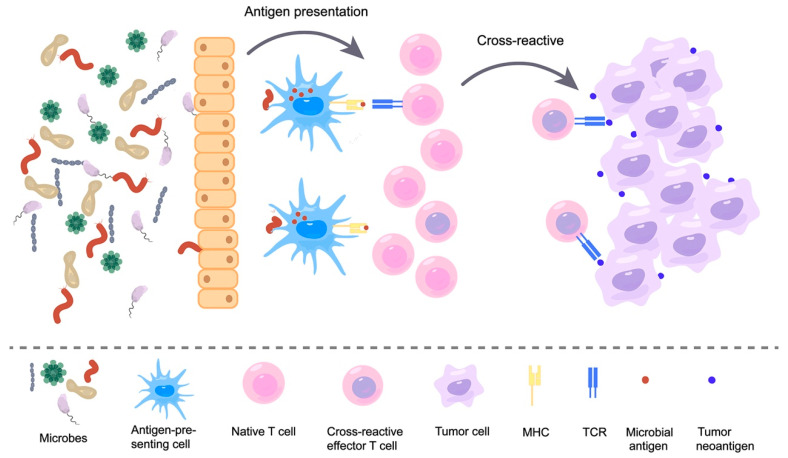
Microbes induce T-cell cross-reactivity by mimicking tumor neoantigens.

These microbial-derived peptides that resemble tumor neoantigens are molecular mimicry, which is a concept that is widely used in the field of autoimmunity [90,91]. Molecular mimicry is the sharing of sequence or structural similarity between foreign antigens and self-antigens; thus, T cell receptors (TCRs) that recognize pathogenic antigens can also recognize self-antigens. In recent years, with the continued discovery of microbial-derived peptides, this molecular mimicry theory has been extended to the field of cancer [92,93,94]. This theory holds that neoantigens that share structural features with microbial antigens are more likely to be immunogenic and recognized by TCR libraries. Some researchers have further indicated how this molecular mimicry affects the immune response. Exposure of the body to microbes produces memory T cells, which further recognize tumor surface antigens homologous to microbial antigens. This recognition results in cross-reactivity, ultimately killing tumor cells (Figure 2) [82]. This theory was confirmed in several studies. For instance, one study found that the phage-encoded TMP peptide expressed in *Enterococcus hirae* (*E. hirae*) has the MHC1-binding epitope TSLARFANI and carries this prophage-containing *E. hirae*-induced T-cell anticancer responses in mice and humans. In patients with kidney cancer and lung adenocarcinoma, another *E. hirae* TMP-derived peptide, KLAKFASVV, was found to potentially elicit an anticancer immune response to the non-mutated tumor antigen KLQKFASTV contained in the GPD1-L protein [78]. This finding demonstrates cross-reactivity between commensal microbial antigens and tumor antigens. It has also been found that SVYRYYGL (SVY), which is expressed in *B. breve* in the intestinal commensal bacteria, is homologous to the neoantigen SIYRYYGL (SIY) expressed in the B16 tumor model. Moreover, mice lacking *B. breve* were found to have reduced SVY-reactive T cells and faster tumor growth compared to mice colonized with *B. breve*. This shows that the neoantigen mimicry of commensal bacteria can stimulate anti-tumor immune responses through T-cell cross-reactivity [77]. Furthermore, the possible role of “molecular mimicry” in anticancer immunity is supported by the identification of sequences highly homologous to immunogenic neoepitopes of CT26 cells in the proteome of specific intestinal flora (the abundance of which directly correlates with tumor regression) of a BALB/c-CT26 cancer mouse model treated with oral *Bifidobacterium* [80]. To evaluate the molecular mimicry theory, a research team engineered *Escherichia coli Nissle* to take on the SIINFEKL epitope (OVA-*E. coli Nissle*). They then orally administered this engineered *E. coli* to C57BL/6 mice. Compared to controls, OVA-*E. coli Nissle* induced OVA-specific CD8^+^ T cells and inhibited the growth of OVA-expressing B16F10 melanoma cells. Next, researchers took a shotgun sequencing of the microbiome. They sequenced the TCR of T cells and demonstrated that the main reason for tumor suppression was mediated by cross-reactive T cells triggered at the intestinal site [95]. These findings confirmed that the microbes can trigger T cell cross-reactivity through their own expression of peptides that are highly similar to tumor antigens and thus affect tumor development.

Although these studies have confirmed that microbial molecular mimicry can inhibit tumor growth through T-cell cross-reactivity, there is still a lack of evidence and studies on whether their presence is universal and whether they have an effective stimulation effect on T cells. It has been shown that tumors are flooded with anti-microbial T cells, such as tumor-infiltrating cytotoxic T lymphocytes (TIL). Moreover, CD8^+^ TILs present in human lung and colorectal cancer are not only specific for tumor antigens but also recognize viral epitopes [96]. Researchers used bioinformatics techniques to compare tumor antigen libraries with intestinal bacteria and viral sequences and found substantial sequence homology between them [85,89]. Furthermore, some investigators have identified peptides from intracellular bacteria in melanoma tumors with the help of HLA peptidomics and 16S rRNA sequencing. They demonstrate that the bacteria that colonize melanoma tumors can enter melanoma cells and that their peptides can be presented on the surface of tumor cells [97]. In a recent study, researchers injected persistently infected cytomegalovirus mouse (MCMV)-derived T cell epitopes into tumors. They found that CMV-specific T-cell responses could be redirected into tumors to stimulate anti-tumor immune responses [98]. It is suggested that viral-derived peptide epitopes can effectively activate anti-tumor T-cell responses.

Individuals are exposed to an environment full of various microbes. These microbes enter the body through a barrier and are recognized by patrolling immune cells, such as antigen-presenting cells. Subsequently, they are presented on MHC. These presented microbial antigens are recognized by T cells and cause T cells to activate and show killing effects. As the picture on the right shows, neoantigens presented on the surface of tumor cells are sometimes highly similar to these microbial antigens. Thus, T cells that recognize microbial antigens can also recognize similar tumor neoantigens, causing T cell cross-reactivity and eventually killing tumor cells.

## 4. Applications of Microbes in the Treatment of Tumor Neoantigens

### 4.1. Application of Microbes in Tumor Neoantigen Vaccines

Tumor neoantigens, with their highly specific expression, have long been considered ideal targets for tumor therapy [99]. In 2017, two articles published in the journal Nature simultaneously reported on the therapeutic role of individualized tumor neoantigen vaccines in human melanoma. One was a 15–30 amino acid peptide mixture vaccine using poly-ICLC (a TLR3 stimulator) as an adjuvant [100], and the other was an mRNA vaccine encoding multiple tumor neoantigen epitopes [101]. Vaccination of melanoma patients had a good therapeutic effect on patients, demonstrating the great potential of personalized neoantigen vaccines in tumor therapy. However, less than 1% of mutant neoantigens in cancer cells can be spontaneously presented to the immune system to elicit an immune response [102]. Therefore, neoantigen vaccines need to be developed with the help of suitable vectors. Efficient tumor vaccines usually require the assistance of immune adjuvants and delivery vectors [103]. Microbes and their components, as natural foreign substances that can enter the host immune system and synergistically promote the immune response, have been widely used as vaccine-delivery vehicles and adjuvants [104]. They have also been explored in the delivery of tumor antigens [16,17]. Several recent studies have found that these microbial components also achieve good outcomes in the delivery of neoantigen vaccines. There is a study in which a tumor neoantigen was incorporated into the vaccine vector of attenuated *Listeria* monocytogenes (Lm). It was found that the vaccine effectively induced activation of specific CD8^+^ T cells and prevented tumor growth [105]. These vaccine delivery vectors derived from microbial components are diverse [106,107]. Here, we focus on neoantigen delivery platforms based on bacterial outer membrane vesicles (OMV) and phages. They have recently achieved good therapeutic outcomes in the application of neoantigen vaccines and are considered to be efficient vaccine vectors [18].

#### 4.1.1. Bacterial Outer Membrane Vesicles and Delivery of Neoantigen Vaccines

OMVs are spherical particles derived from Gram-negative bacteria [108]. These vesicles contain many immunogenic substances from parental bacteria and have the ability to activate the innate immune system. Furthermore, they can be genetically engineered to express selected antigens, thus having great potential for vaccine production [109]. Recently, researchers have used a “plug-and-display” strategy to fuse foreign tumor neoantigens with Cly-A, a protein commonly found on the surface of OMVs, using recombinant gene technology to present exogenous antigens on the surface of OMVs. It was demonstrated that this tumor antigen displayed on the surface of OMVs could induce T cell-mediated specific anti-tumor immunity. They also developed a bioengineered tumor antigen display system for OMVs capable of displaying multiple antigens simultaneously, which provides great value for the development of individualized tumor vaccines [110]. There is also an mRNA vaccine platform based on the same “plug-and-display” strategy. By genetic engineering, the archaeal RNA-binding protein L7Ae was fused to the C-terminus of the OMV surface protein ClyA. Then, the mRNA was modified in vitro, ultimately designing an OMV that can effectively display mRNA antigens. Moreover, pathogen-associated molecular patterns (PAMPs) in OMVs also enhance the activation effect of antigen-specific T cells and suppress tumor development [111]. Meanwhile, an in situ vaccine study of OMVs has shown an alternative treatment option. Photothermal therapy (PTT) can activate tumor-specific T cells by releasing tumor antigens. Here, researchers constructed an OMV in situ vaccine, OMV-Mal, that captured these tumor antigens. They demonstrated that this vaccine could effectively deliver tumor antigens to dendritic cells and ultimately activate anti-tumor immune responses with the help of OMVs [17]. OMVs can integrate vectors and adjuvants in tumor vaccines to activate multiple immune signaling pathways to their own advantage [112], providing a significant role in tumor neoantigen vaccine therapy value.

#### 4.1.2. Phage and Neoantigen Vaccine Delivery

In addition to these bacterial-derived components, phages are also considered an ideal vehicle for neoantigen vaccines due to their characteristics [113]. The phage display technique was first introduced by Smith et al. in 1985 [114], which allows the expression of a variety of exogenous peptides on the surface of phages, targeting a variety of molecules [115,116]. Phage particles can also act as a foreign substance recognized by the body’s immune system and presented by antigen-presenting cells to MHC I or MHC II molecules, inducing specific humoral or cellular immunity. This makes phages attractive vaccine carriers [117]. In recent years, the display of antigen peptides on the surface of phages with the help of this technique has become increasingly considered an effective cancer vaccine delivery strategy [118]. Many researchers have successfully constructed vaccines expressing tumor antigens with the help of phages. For example, a recombinant T7 phage vaccine expressing a new epitope of the B16-F10 melanoma cell mutant protein was constructed. And it was found that vaccination with these vaccines induced B-lymphocyte responses in mice and the effective production of specific antibodies [119]. Another study constructed a λ-phage vaccine displaying the HER2/neu-derived peptide GP2, which was vaccinated in a BALB/c mouse transplantation tumor model, showing that this fusion peptide-expressing phage nanoparticles induced a robust cytotoxic T lymphocyte (CTL) response [120]. These achievements demonstrate the great progress of phage display technology in the development of neoantigen vaccines, yet the development of efficient vaccine platforms remains a great challenge. Recently, some researchers have developed several efficient neoantigenic vaccine presentation platforms using phage display technology. Li W. et al. designed an antigen peptide vaccine delivery system based on P22 virus-like particles (VLPs). They prepared two types of vaccine particles (VLP-OVAB and VLP-OVAT) by presenting the B-epitope and the T-epitope of ovalbumin (OVA) in VLPs that were selected from the P22 phage. In their experiments, VLP-OVAB induced high titer antibody levels (5.0 × 10^5^) and effectively activated CTL responses by cross-presentation, while VLP-OVAT induced strong immune activation and immune memory and remarkably inhibited tumor growth [121]. Another study developed a vaccine delivery platform (HMP@Ag) that can deliver personal tumor antigens by M13 phage using a chemical approach to adsorb various antigen substances onto M13 phages by electrostatic. When the mice were vaccinated subcutaneously, such a hybrid M13 phage carrier could effectively promote antigen delivery and cross-presentation and activate cytotoxic CD8^+^ T cells. Furthermore, combining the HMP@Ag vaccine with immune checkpoint blockade (ICB) treatment can trigger a robust and specific anti-tumor immune response in several tumor models [122].

This microbial-derived biological nanoparticle material has demonstrated great application in the development and delivery of neoantigen vaccines due to its ability to carry naturally occurring PAMPs and the ease of genetic engineering modifications.

### 4.2. Microbes Improve Treatment of Immune Checkpoint Inhibitors (ICIs) by Influencing Neoantigens

#### 4.2.1. Gut Microbes Play a Role in the Treatment of ICIs

With the rise of tumor immunotherapy, ICB therapy has become a major weapon in the fight against cancer over the past decades [123]. ICIs activate T cells and promote their anti-tumor function by targeting and blocking PD-1, PD-L1, CTLA-4, LAG3, and other immunosuppressive targets [124,125]. Immunotherapy with the help of ICIs has led to breakthroughs in the treatment of a variety of malignancies [123,126,127,128]. Additionally, a number of ICIs have been approved for clinical cancer treatment [129]. However, ICB therapies still have limitations, as they show low response rates (10–30%) in most cancer treatments (10–30%) [123] and present drug resistance [130,131], and only a small proportion of patients benefit from ICB. Therefore, it is necessary to further investigate the mechanism of ICB and find ways to improve the effectiveness of immune checkpoint blockade therapy.

Studies in recent years have found that the gut microbiota that interacts with the organism plays an important role in the treatment of ICIs. This influence has been confirmed in many cancer models. Two studies in 2015 in mouse models of melanoma first showed that gut microbiome composition can modulate the host immune system and influence the efficacy of anti-PD-L1 and anti-CTLA-4 treatments [132,133]. Influencing the host microbiota by administering antibiotics demonstrated that the intestinal microbiome significantly influenced the outcome of PD-1 blockade in mice and patients with non-small cell lung cancer (NSCLC) and kidney cell carcinoma (RCC) [134]. Analyzing the fecal microbiome of patients with hepatocellular carcinoma (HCC) during anti-PD-1 immunotherapy by macrogenomic sequencing revealed microbiome-specific changes and suggested an association between the gut microbiome and anti-PD-1 immunotherapy [135]. Immunotherapy with anti-PD-1 in an antibiotic-treated colorectal cancer (CRC) mouse model revealed that antibiotic injection counteracted the efficacy of the PD-1 antibody. Additionally, it was demonstrated that gut microbiome changes affected the tumor’s immune microenvironment [136]. All these studies illustrate the critical role of microbes in ICB therapy. Most of these microbes are gut microbes because the gastrointestinal tract involves the entire digestive system and has an abundance of microbes [137]. Moreover, the gastrointestinal tract is relatively easy to study, allowing interventions by simple means [138,139]. Gut microbes not only inhibit tumor growth but also promote tumorigenesis and progression. Currently known oncogenic gut bacteria include *Salmonella typhi* [140] and *Helicobacter* spp. [141]. It has been found that certain microbes can also block the body’s anti-tumor immune function and form a pro-inflammatory microenvironment that contributes to cancer progression. It is commonly believed that dysregulation of gut microbiota homeostasis is associated with cancer development and progression [142]. This dual role of gut microbes is due to the complexity of gut microbial species, with different species causing different consequences. For example, the antigenic peptide produced by the microbes described earlier causes T-cell cross-reactivity, and whether the immune response provoked by this antigenic peptide is pro- or anticancer depends on the specific peptide. Suppose it so happens that this antigenic peptide has similarities to tumor antigens. In that case, it may kill tumor cells, while other microbial peptides may cause host immune suppression or disruption of the immune system, with the disease progressing in a worsening direction [143]. Here, we focus on those microbes that may have a positive effect on cancer therapy.

Comprehensive analysis of 16S rRNA and shotgun metagenome sequencing of the gut microbiome revealed a significantly higher (2.5-fold) ratio of *Prevotella*/*Bacteroides* in the gut microbiome of gastrointestinal cancer patients who showed a better response to ICIs treatment [144]. In addition to these, a correlation between the abundance of *Akkermansia muciniphila* and the response to treatment with ICIs was found in the intestinal bacteria of patients with NSCLC and RCC [134]. In the analysis of the bacteria in melanoma patients, symbiotic *bifidobacteria* were found to play an important role in the anti-tumor process [133]. In patients with metastatic melanoma treated with anti-CTLA-4 checkpoint inhibitors, it was observed that patients enriched with *Faecalibacterium* and other *Firmicutes* had a better therapeutic effect [145]. In addition to the gut microbiome, microbiota at other sites, such as the skin and liver, showed a correlation with ICB therapy [146]. In conclusion, tumor-related microbes play an encouraging role in ICB therapy.

#### 4.2.2. Microbes Enhance the Therapeutic Effect of ICIs by Molecular Mimicry

How exactly do these microbes affect ICB therapy? Why are they performing well in the treatment of ICIs? There is no conclusive answer yet. We know that a successful anti-tumor response in ICB therapy relies on the activation and proliferation of specific T cells. It is crucial to effectively activate tumor-specific T cells that target tumors during the whole treatment process [147]. The reasons for the poor therapeutic effect of ICIs can be briefly summarized into three categories: insufficient anti-tumor T cell production, insufficient tumor-specific T cell function, and memory T cell formation [148]. Microbial modulation of ICB therapy is ultimately achieved by affecting these immune cells and thus affecting tumor therapy. In 2016, Laurence et al. synthesized the findings at that time and proposed two microbial mechanism hypotheses for tumor immune surveillance that helped us better understand the role of microbes in ICB therapy. One mechanism is the antigen pathway, in which microbial antigens that are highly similar to tumor antigens activate specific anti-tumor T cells by affecting the immune system and generating T cell cross-reactivity. Another mechanism is the non-antigen pathway, in which these microbes regulate immune tonus through their own PAMPs, producing a series of metabolites such as interferons and cytokines to modulate T cell anti-tumor activity [92,149]. In the treatment of ICIs, microbes can influence tumor therapeutic effects through both of these pathways.

In recent years, an increasing number of studies have provided evidence for the first antigen mimicry mechanism. Here, we focus on how microbes can influence immune checkpoint blockade therapy through tumor antigen mimicry. Tumor neoantigens, recognized by the immune system as external peptides, possess distinct specificity and immunogenicity. These neoantigens can be selectively engaged by T cells, which holds significance in the context of ICI treatment. The absence of these neoantigens can lead to therapy resistance [131]. ICB therapy can effectively activate tumor neoantigen-specific T cells for better anti-tumor effects [11,150]. At the same time, microbes can help activate specific T cells through antigen mimicry, enhancing the therapeutic effect of ICIs. In 2020, researchers found that the commensal bacterium *Bifidobacterium breve* (*B. breve*) contains a peptide (SVY) that is highly similar to a melanoma-specific epitope. They also found that mice lacking *B. breve* had fewer SVY-reactive T cells and faster tumor growth. They further demonstrated that *B. breve* can specifically kill tumor cells by causing T-cell cross-reactivity through antigen mimicry [77]. In addition, previous studies found that *B. breve* from gut microbes plays an important role in the anti-tumor process in melanoma patients and can combine with PD-L1 antibodies to produce a good therapeutic effect in mice [133]. These two studies provide us with a thought that perhaps *B. breve* enhances the anti-PD-L1 therapeutic effect of melanoma by T-cell activation through molecular mimicry. In addition, another study identified a tail length tape measure protein (TMP) of prophage in the bacteriophage *Enterococcus hirae* genome that could bind MHC I epitopes and induce memory CD8^+^ T-cell responses, which in turn cross-react with cancer antigens. Immunotherapy with anti-PD-1 induced TMP-specific CD8^+^ T cell responses and showed that the presence of the bacteriophage *Enterococcus hirae* and expression of TMP cross-reactive antigens correlated with the long-term efficacy of PD-1 blockade therapy in patients with kidney and lung cancer [78]. These examples suggest that microbial-associated peptides can activate anti-tumor T cells through cross-reactivity to target tumor cells with neoepitopes in ICB therapy.

#### 4.2.3. Use of Microbes to Enhance the Treatment of ICIs

The hypothesis about the effect of microbes through antigen mimicry has gradually become convincing in recent years [149,151]. High tumor mutation burden (TMB) significantly improves the ICI’s therapeutic effect, as demonstrated in many studies [152,153]. Although tumor-specific antigens are mechanistically thought to promote ICB therapy, they lack further therapeutic potential. In contrast, the microbiome can be more dynamically regulated to influence T-cell responses against tumor-specific epitopes and thus improve the efficacy of ICIs [81]. And interventions such as probiotic supplementation and increased microbial diversity could be considered rational therapeutic approaches for clinical investigation [13].

Fecal microbiota transplantation (FMT) therapy has shown promising effects in recent studies. FMT therapy allows the transfer of the entire gut microbiota from one host to another, usually transferring the gut microbiota from patients who have responded to ICI therapy to immune-tolerant patients [154]. Recently, researchers have achieved positive outcomes in phase I clinical trials by using FMT treatment. It was shown that after treatment with FMT and anti-PD-1 re-induction, three out of ten patients with melanoma lacking responsiveness to anti-PD-1 therapy had a decrease in tumor volume. Notably, two of these had complete remission and one had partial remission [155]. Another clinical trial evaluated the safety and efficacy of FMT in combination with anti-PD-1 therapy in patients with PD-1 refractory melanoma. In this trial, six of fifteen patients demonstrated a beneficial response, with three patients achieving remission and three patients having stable disease [156]. In both trials, a good safety profile was demonstrated, showing great value in clinical treatment. In addition to this, there are also many clinical trials of combined treatment with FMT and ICIs currently underway [157].

Furthermore, modifying microbes via genetic engineering or using their own characteristics and then delivering them back into the body to work is also a convenient and feasible treatment modality [158]. Through genetic engineering, these microbes can carry various target genes to improve the therapeutic effect of ICIs. Among them, the combination therapy of oncolytic viruses (OVs) and ICIs has shown good promise. OVs have good properties that cause the lysis of tumor cells to release tumor neoantigens and activate specific T cells, and they are widely used in immunotherapy [159]. In parallel, clinical trials using OVs in cancer therapy have highlighted the importance of combining them with checkpoint inhibitors [160,161]. For example, local infection with OVs in tumor models with resistance to ICIs therapy revealed that OV infection triggered activation of T cells targeting tumor neoepitopes, significantly eliminating systemic resistance to PD-1 immunotherapy and improving the elimination of disseminated lung tumors [162]. In addition to those, OV can be designed to express various immunomodulatory genes [163]. Many combination therapies have been developed, such as when researchers engineered a new oncolytic herpes simplex virus (oHSV) expressing a single-chain antibody against PD-1 (scFvPD-1) and evaluated its efficacy against glioblastoma (GBM). This confirms that it induced durable anti-tumor responses in a preclinical mouse model of GBM [164]. Also, researchers generated an engineered OV co-expressing PD-L1 inhibitor and a genetically modified granulocyte-macrophage colony-stimulating factor (GM-CSF). When these engineered OVs were injected into tumors, they overcame PD-L1-mediated immunosuppression during both the priming and effector phases, activated a systemic T-cell response, and led to an effective rejection of both virus-injected and distant tumors [165].

## 5. Conclusions and Perspectives

Immunotherapy targeting tumor neoantigens has attracted many researchers in recent years and is considered to have great promise in cancer immunotherapy [166]. However, neoantigen-driven immunotherapies still face great challenges in clinical application [167], such as an insufficient number of neoantigens with immunogenicity and inadequate activation of specific T cells targeting neoepitopes. In the early days of neoantigen identification, researchers mainly targeted single nucleotide-producing mutations due to technical limitations. After decades of development, the content of neoantigens has been continuously expanded, and more and more previously unnoticed neoepitope types have been classified as tumor neoantigens [4]. The exploitation of potential neoantigen pools, especially those with high immunogenicity, has been a constant research direction in related fields. Abnormalities can occur during gene expression, transcription, translation, and post-translational modifications, which can lead to the formation of neoepitopes. Although some studies have shown that some classes of neoantigens are more protective, there are no comprehensive studies to determine which are the most effective neoantigens [8]. In recent years, neoepitopes that are highly similar to microbial-derived peptides have been found to exhibit good immunogenicity in studies [56,77]. Some researchers have introduced the concept of homology with pathogenic peptides in the comprehensive assessment models constructed for the immunogenicity of neoantigens [168]. Homology comparison with foreign microbial peptides may be a feasible approach for the identification of neoantigens.

We are exposed to an environment populated by microbes. These microbes not only regulate the health of the body but also influence the onset and development of disease. Recent advances in oncology have listed the polymorphic microbiome among the fourteen features of malignancy [169]. Several studies have shown that the flora in the body plays an important role in immunity [13]. For example, in the microbiome-enriched gastrointestinal tract, it has been found that the efficacy of ICB therapy for malignant tumors is closely linked to intestinal flora. And some specific microbial species are highly correlated with the effectiveness of therapies such as anti-PD-1/PD-L1 [132,133]. In addition, probiotic supplementation or the use of FMT therapy during cancer immunotherapy can also help treatment [154,155,156]. Although the exact mechanism of microbial influence on immunotherapy remains unclear, recent studies have found that microbes, through their own peptides that are highly similar to tumor antigens, may be able to influence the immune system. This generates a T-cell cross-reaction, activating specific anti-tumor T cells. Meanwhile, some studies have found that these microbial antigenic peptides, which are similar to tumor neoantigens, play an important role in immune checkpoint therapy [77,78]. These examples remind us that perhaps the combination of immune checkpoint inhibitors with HLA that mimic tumor antigen-like peptides will improve therapeutic and prognostic outcomes.

Currently, many researchers are working on the development of new antigenic vaccines for cancer. Additionally, many vaccines are being evaluated in early clinical trials, such as synthetic long peptide (SLP) vaccines, dendritic cell (DC) vaccines, and nucleic acid vaccines [7]. The success of cancer vaccines is influenced by many factors, among which the superiority of the vaccine platform is an important reason [170], and the development of a simple and efficient cancer vaccine platform is necessary. As described above, microbes such as bacteria and phages are structurally simple. They can be modified by genetic engineering and have exogenous immunogenicity, which plays a significant role in cancer vaccine development and is a good delivery vehicle [18,106,111,120]. They have great potential for neoantigen vaccine development.

Regardless of which therapeutic technique is used, the fundamental aim of cancer immunotherapy is to regulate the patient’s own immune system, causing immune cells to attack the tumor [2,12]. In practical research and applications, neoantigens with high immunogenicity and efficient and convenient therapeutic approaches are worth continuously exploring. The microbes that fill our lives are closely linked to the development of cancer. As mentioned above, microbes play an important role in the therapeutic process against tumor neoantigens [16,17,104]. There is evidence that tumor neoantigens homologous to peptides from microbes could better activate anti-tumor immune responses during therapy [77,78]. However, the evidence is currently scarce, and more studies are needed to reveal confirmation in the future. It is also important to consider how to use this property to treat tumors. The good results triggered by FMT in the treatment of ICIs may be related to this effect. Some specific microbial species have been identified to play a role in this process [133,145,154,155], and whether these microbes can be better used in the treatment of ICIs still needs to be explored. In addition, developing more microbial tools, such as vaccine delivery vectors, adjuvants, etc., will also facilitate immunotherapy against neoantigens in tumors.

## Figures and Tables

**Table 1:** Tumor neoantigens that share homologs with microbial peptides.

Neoantigen Peptide	Microbial Peptide	Tumor Type/Species	Microbial Species	Reference
NLLGRNSFK	LLGRNSFEV	Pancreatic ductal adenocarcinoma/Human	*Homo sapiens*	[56]
QEFENIKSY	QRFHNIRGR	Pancreatic ductal adenocarcinoma/Human	*Human papillomavirus*	[56]
GIICLDYKL	TMGVLCLAIL	Pancreatic ductal adenocarcinoma/Human	*Dengue virus*	[56]
LLLMSTLGI	LLMGTLGIV	Pancreatic ductal adenocarcinoma/Human	*Human papillomavirus*	[56]
QTYQHMWNY	AFWAKHMWNF	Pancreatic ductal adenocarcinoma/Human	*Hepatitis C virus*	[56]
LPRQYWEAL	KLLPEGYWV	Pancreatic ductal adenocarcinoma/Human	*Francisella tularensis*	[56]
RPQGQRPAL	SPRGSRPSW	Pancreatic ductal adenocarcinoma/Human	*Hepatitis C virus*	[56]
RVWDIVPTL	KPWDVVPTV	Pancreatic ductal adenocarcinoma/Human	*Dengue virus*	[56]
SIYRYYGL	SVYRYYGL	Melanomas/Mouse	*Bifidobacterium breve*	[77]
GSLARFRNI	TSLARFANI	MCA205 sarcomas and TC1 lung cancers)/Mouse	*Siphoviridae phages*	[78]
TLAGFWARL	RLAGFFPRL	CT26 M12/Mouse	*Ruminococcaceae*	[80]
PGPWRSGRLL	LGPWRSGGVL	CT26 M19/Mouse	*Bacteroidales/Prevotella/* *Muribaculacee*	[80]
SMPGPWRSG	SLPGSWRSL	CT26 M19/Mouse	*Bacteria*	[80]
YIALVDKNI	YIALFDGFI	CT26 M39/Mouse	*Duncaniella/Bacteroides/* *Bacteroidales*	[80]

## Data Availability

Not applicable.
